# An Essay on Syphilis

**Published:** 1821-03-01

**Authors:** 


*An Essay on Syphilis; submitted, by Authority of the President and his Council, to the Examination of the Royal College of Surgeons of Edinburgh, when Candidate for admission into their Corporation.* By GEORGE BALLINGALL, M.D. F.R.S.E. F.R.C.S.E. and late Surgeon to His Majesty's Thirty-third Regiment. Octavo, pp. 36. Edinburgh, 1820.

BY observation of its title-page, our readers will readily discern the nature and object of this probationary essay. On a former occasion (*Quarterly Series of this Journal, No. V. p. 26*) we introduced its author to the profession, by an analytical review of his able and useful Observations on Indian Dysentery, and other intertropical diseases. Since publishing that work, he has obtained, in 1819, the degree of Doctor in Medicine from the University of Edinburgh, after defending the doctrines of an Inaugural Dissertation on Sanguineous Apoplexy. As we deem the sentiments of such a man to be at all times worthy of respect, we assume the merit of being profitably employed, when we endeavour to disseminate his views of an important practical question.

Dr. Ballingall treats his subject under the two-fold arrangement of—Preliminary Remarks on the Use of Mercury in Syphilis—and Observations on the Treatment of the primary Symptoms of the Disease.

I. Strengthening his opinions by the authority of Mr. Pearson, Dr. Curry, Mr. B. Bell, Dr. Watt, Mr. Carmichael, Mr. Matthias, and other eminent names, Dr. Ballingall declares himself an adherent to the mercurial treatment of syphilis; but, at the same time, acknowledges this to be the only mode of treating it, whereof he has had extensive experience.

"Upon referring" says he, p. 7, 8, "to a register of sick in my possession, which was kept at Masulipatam in the East Indies, from the 15th of March, 1810, to the 17th of February, 1811, I find that, out of a detachment of the 2nd battalion of the Royals, consisting of about *five hundred* men, *eighty-six* venereal cases were admitted into hospital; that these were, upon an average, *twenty-two days* each, under treatment; and, that not more than *seven* secondary cases could possibly have occurred.*

"This is the only period of my experience in the treatment of syphilis, of which any documents in my possession, enable me to speak with precision: but, when I recollect that nothing peculiar was observed, during that period, either in the appearance of the disease, in the progress of the cures, or in the number of secondary cases occurring, I cannot but look on the above, with some degree of confidence, as a tolerably exact estimate of the result of all my observations on this point, and the estimate must necessarily be an unfavourable one, from my having included, under the head of secondary cases, all those whose names occur a second time in the register, although, of these, it is probable some were re-admitted with recent infections, instead of secondary symptoms. When I consider again, that, for many years of my life, I was in the habit of seeing, at least, from *ten* to *twenty* venereal cases daily; that these cases were almost uniformly treated with mercury; that the cures were as speedy and the relapses as few as I have stated them to be at Masulipatam; and that, in the whole course of my observation, I have seen only *one* man die of this disease, I must necessarily look upon mercury as more uniformly successful in the cure of syphilis, than any other remedy in any other disease with which I am acquainted."

II. As the more immediate business of his essay, Dr. Ballingall now proceeds to consider the primary symptoms of syphilis—chancres and buboes.

Assuming the Hunterian characteristic of chancre—"circumscribed hardness of the edge and base"—as liable to vary in degree, and that this hardness may be shaded down until it becomes nearly indistinguishable, Dr. Ballingall, p. 12,

---

* "The proportion of venereal cases which occurred at Masulipatam, was greatly below what I was accustomed to meet with in India, owing to the vigilance and activity of Mr. Annesley, the garrison-surgeon, who superintended the Lock Hospital."

advises our not confining "ourselves too rigidly to this definition in deciding upon the mode of cure, particularly if we are to exclude from the beneficial operation of mercury all ulcerations of the genitals, which do not possess the Hunterian character of chancre; for my experience," says he, "convinces me that the cure of many of these ulcerations will be expedited by mercury, when a circumscribed hardness of the edge and base does not exist in any remarkable degree."

Dr. Ballingall, p. 13, follows Mr. B. Bell "in extending the appellation of chancre to sores in the genitals, which offer a considerable variety in appearance." His treatment, of course, is the mercurial, under such modifications as circumstances may indicate. He advises confinement to the house, in all cases when practicable—to heal the chancrous ulcerations with the least possible delay—to keep them clean by frequent ablution, and dressing with dry lint—to introduce mercury into the system in moderate proportion, by means of the blue pill, by inunction, or by the mineral combined with opium—and, when there is evidence of the system being moderately impregnated, to touch the sores with lunar caustic, to dress them with ung. oxyd. hydrarg. rubr. or preferably with ung. subacet. cupri, or to wash them with lot. hydrarg. submur. nigr. attention being paid, at the same time, to the variations of constitutional symptoms.

Dr. Ballingall's description of the venereal bubo, and his history of its symptoms, its diagnosis, its treatment, are luminous and accordant with the experience of intelligent observers. His treatment is simple, but decisive. "Every part of the antiphlogistic regimen," says he, p. 22, "is to be combined with the use of mercury, and the assiduous employment of every local means," such as topical leechings, sedative and astringent lotions, particularly those of the acetate of lead, "to promote the dispersion of the tumour."

When the inflammation is violent in plethoric habits; and, particularly, as frequently happens, when "there is a fiery erysipelatous appearance on the surface of the tumour, general blood-letting," in Dr. B's estimation, p. 22, "is the only means which can effectually avert suppuration." When he meets with "tumours of a mixed nature, evidently originating from a venereal infection, while, in their progress they seem more a-kin to the scrofulous bubo, remaining for days, and sometimes, for weeks quite stationary, without shewing a decided tendency either to resolution or suppuration," he forthwith directs blisters to be applied to their surfaces. In a majority of such cases, he declares, p. 24, his confidence in the blisters' being able to "procure discussion of the swellings, and in all of them, certainly to expedite their termination in resolution or suppuration."—" This is a practice," he goes on to say, p. 24, " by no means so general as it ought to be, and to some I know that it is altogether new. —It is one which I have been in the habit of using very extensively ever since I entered the army, now nearly fifteen years ago, and it may be supposed I am not the less inclined to persevere in it, from finding it recently recommended by a gentleman of Mr. Carmichael's talents and experience." 'The buboes in this form of venereal disease,' says Mr. C. p. 21,* 'are often remarkably hard and indolent, evincing neither a tendency to disperse nor to suppurate. In such cases, the greatest advantage may be derived from the repeated application of blisters to the indurated bubo, which soon either causes the dispersion or the suppuration of the tumour.'

Failing to avert suppuration, Dr. Ballingall renounces the discutient treatment, and endeavours to promote that process by an assiduous use of fomentations and warm emollient cataplasms frequently renewed. Instead of opening a bubo by the caustic, he prefers evacuating its contents by a free incision of the teguments with a common or abcess lancet, and " nothing," says he, p. 25, "should induce us to be too sparing in the extent of the opening, which frequently leads to the formation of sinuses requiring renewed operations, and unnecessarily protracting the sufferings of the patient." He advises, p. 27, the passing of a small seton through the base of extensive buboes when they advance to suppuration, while their integuments remain firm and little discoloured on the surface. In one case of open bubo, with a sinus, he accomplished a cure contrary to his expectations, by injecting a solution of the sulphate of iron, by which "the sinus," p. 27, " was completely and firmly healed in a few days."

Dr. Ballingall concludes his observations on the treatment of venereal chancres and buboes, with two extracts from his friend Dr. Hennen's valuable work on Military Surgery. " The Doctor," says he, p. 29, " brings forward some very striking instances of the sufficiency and permanency of cures effected without mercury, while at the same time, he is far from denying the utility of this medicine in some cases of syphilis, as will be seen by the extract at page 285 of our second Number of this Series. Fortunately, for the good of mankind and the honour of our profession, this question of the possibility of eradicating syphilis, with as much or more

---

* Carmichael's Observations on the Symptoms and Specific Distinctions of Venereal Diseases, 8vo. London, 1818.

convenience and perfectness, by a non-mercurial as by the mercurial treatment, has hitherto been discussed with the moderation, the generous zeal, and the dignity, by which the investigations of science and philosophy ought at all times to be distinguished. Many facts drawn from the repositories of actual observation have, in consequence, been promulgated by the advocates of either practice, in support of the doctrines which their reasonings are destined to maintain. Nevertheless, experience may, in the end perhaps, induce us to prefer a modification of the rival precepts—sometimes the mercurial, sometimes the non-mercurial, generally a treatment comprizing the principles of both—a modification, such as circumstances may require and prudence discriminate. By such mixed treatment, we mean an exhibition of mercury, as a balancer of the vital functions, as a reducer of febrile and inflammatory action, in co-operation with the non-mercurial treatment strictly so designed.

Among the leaders in these interesting disquisitions may be ranged, Mr. Allan* of Edinburgh, and Mr. S. Cooper† of London; the former a naval, the latter a military, surgeon, each of whom has favoured the profession with his sentiments on this question, so intricate in its nature, and so important in its results. Mr. Allan, p. 194—196, 205—207, 218—19, gives a decided preference to the mode of treating syphilis with mercury; while Mr. Cooper's opinions, Vol. I. p. 354—56, favour very strongly, but temperately, the non-mercurial practice. Having, however, devoted an ample space in our second No. to the exposition of this grave subject, we shall not, at present, resume it, but complete this sketch with Dr. Ballingall's manly and dispassionate avowal.

"I am still far," he declares, p. 5, "from being disposed to undervalue the labours of those eminent men who have recently written in favour of the non-mercurial treatment of syphilis: I fully appreciate the benefits accruing to scrofulous and phthisical patients, from the proof which has been given of the possibility of curing the disease without mercury; and I am most ready to admit, that the recent discussions upon this subject, have been of infinite advantage, both to the profession and the public, by restricting the use of so

---

* A System of Pathological and Operative Surgery, founded on Anatomy; illustrated by Drawings of Diseased Structure, and Plans of Operations; by Robert Allan, F.R.C.S. E. & L. 8vo. Edinburgh, 1819 Vol. I. pp. 496.

† The First Lines of the Practice of Surgery; designed as an Introduction for Students, and a concise Book of Reference for Practitioners; by Samuel Cooper, late Surgeon to the Forces, &c. Two Vols. 8vo. London, 1820.

powerful a remedy, which, like all others, in the same proportion that it is useful under judicious administration, is capaple of doing mischief by its unnecessary, ill-timed, or injudicious employment."

This little tract, upon the whole, is very creditable to Dr. Ballingall's good sense and accurate observation; nor does it detract from the reputation which this gentleman has well and honourably earned by his very valuable practical work on Indian diseases.

